# Greigite: a true intermediate on the polysulfide pathway to pyrite

**DOI:** 10.1186/1467-4866-8-1

**Published:** 2007-03-21

**Authors:** Stefan Hunger, Liane G Benning

**Affiliations:** 1 Earth and Biosphere Institute, School of Earth and Environment, University of Leeds, Leeds, LS2 9JT, UK

## Abstract

The formation of pyrite (FeS_2_) from iron monosulfide precursors in anoxic sediments has been suggested to proceed via mackinawite (FeS) and greigite (Fe_3_S_4_). Despite decades of research, the mechanisms of pyrite formation are not sufficiently understood because solid and dissolved intermediates are oxygen-sensitive and poorly crystalline and therefore notoriously difficult to characterize and quantify.

In this study, hydrothermal synchrotron-based energy dispersive X-ray diffraction (ED-XRD) methods were used to investigate *in situ *and in real-time the transformation of mackinawite to greigite and pyrite via the polysulfide pathway. The rate of formation and disappearance of specific Bragg peaks during the reaction and the changes in morphology of the solid phases as observed with high resolution microscopy were used to derive kinetic parameters and to determine the mechanisms of the reaction from mackinawite to greigite and pyrite.

The results clearly show that greigite is formed as an intermediate on the pathway from mackinawite to pyrite. The kinetics of the transformation of mackinawite to greigite and pyrite follow a zero-order rate law indicating a solid-state mechanism. The morphology of greigite and pyrite crystals formed under hydrothermal conditions supports this conclusion and furthermore implies growth of greigite and pyrite by oriented aggregation of nanoparticulate mackinawite and greigite, respectively. The activation enthalpies and entropies of the transformation of mackinawite to greigite, and of greigite to pyrite were determined from the temperature dependence of the rate constants according to the Eyring equation. Although the activation enthalpies are uncharacteristic of a solid-state mechanism, the activation entropies indicate a large increase of order in the transition state, commensurate with a solid-state mechanism.

## Background

The formation of pyrite is an important geochemical pathway linking the global biogeochemical cycles of iron, sulfur and carbon in anoxic sediments [1-3]. Furthermore, chemical reactions involved in pyrite formation have important implications for the fate and mobility of toxic [4,5] and radioactive [6] metals in near-surface environments. Over the past half century, the formation of pyrite has been studied extensively at low temperatures and several pathways have been proposed [1,7-10]; yet the mechanisms of pyrite formation in anoxic sediments and the chemical conditions favoring its formation and stability are still not fully understood.

It was recognized early that the formation of pyrite from iron monosulfide precursors in anoxic sediments required an oxidant [1]. In one of the first systematic laboratory investigations of pyrite formation, Berner [1] found that zerovalent sulfur dissolved as polysulfides oxidized iron monosulfide and lead to the formation of pyrite at 65°C (Equ. 1). In addition, he found that the so formed pyrite grains had similar morphologies to natural pyrite framboids and suggested that reaction (1) may thus play a crucial role in most sedimentary environments.

FeS + S^0 ^→ FeS_2 _    (1)

Drobner and coworkers [11] and later Rickard [10,12,13] proposed that hydrogen sulfide can act as an oxidant of iron monosulfide, yielding pyrite and hydrogen gas. Rickard and Luther concluded from polarographic results [7] that aqueous iron monosulfide complexes in equilibrium with the solid phase react with hydrogen sulfide in solution, producing pyrite via a dissolution/re-precipitation pathway [10] (Equ. 2).

FeS→FeS(aq)→−H2H2SFeS2(s)     (2)
 MathType@MTEF@5@5@+=feaafiart1ev1aaatCvAUfKttLearuWrP9MDH5MBPbIqV92AaeXatLxBI9gBaebbnrfifHhDYfgasaacH8akY=wiFfYdH8Gipec8Eeeu0xXdbba9frFj0=OqFfea0dXdd9vqai=hGuQ8kuc9pgc9s8qqaq=dirpe0xb9q8qiLsFr0=vr0=vr0dc8meaabaqaciaacaGaaeqabaqabeGadaaakeaacqqGgbGrcqqGLbqzcqqGtbWucqGHsgIRcqqGgbGrcqqGLbqzcqqGtbWudaWgaaWcbaGaeeikaGIaeeyyaeMaeeyCaeNaeeykaKcabeaakmaaxacabaWaaCbeaeaacqGHsgIRaSqaaiabgkHiTiabbIeainaaBaaameaacqqGYaGmaeqaaaWcbeaaaeqabaGaeeisaG0aaSbaaWqaaiabbkdaYaqabaWccqqGtbWuaaGccqqGgbGrcqqGLbqzcqqGtbWudaWgaaWcbaGaeGOmaiJaeiikaGIaem4CamNaeiykaKcabeaakiaaxMaacaWLjaWaaeWaaeaacqaIYaGmaiaawIcacaGLPaaaaaa@4EE8@

Schoonen and Barnes [8] and Luther [7] have suggested that solid FeS reacts with adsorbed polysulfide via a cyclic intermediate and a combined nucleophilic/electrophilic attack to nucleate pyrite. Luther also proposed from his polarographic results, that a dissolved FeSH^+ ^complex reacts in a similar fashion with polysulfide, nucleating pyrite from solution (Equ. 3) [7].

FeS→FeS(aq)→−Sx-12-Sx2-FeS2(s)     (3)
 MathType@MTEF@5@5@+=feaafiart1ev1aaatCvAUfKttLearuWrP9MDH5MBPbIqV92AaeXatLxBI9gBaebbnrfifHhDYfgasaacH8akY=wiFfYdH8Gipec8Eeeu0xXdbba9frFj0=OqFfea0dXdd9vqai=hGuQ8kuc9pgc9s8qqaq=dirpe0xb9q8qiLsFr0=vr0=vr0dc8meaabaqaciaacaGaaeqabaqabeGadaaakeaacqqGgbGrcqqGLbqzcqqGtbWucqGHsgIRcqqGgbGrcqqGLbqzcqqGtbWudaWgaaWcbaGaeeikaGIaeeyyaeMaeeyCaeNaeeykaKcabeaakmaaxacabaWaaCbeaeaacqGHsgIRaSqaaiabgkHiTiabbofatnaaDaaameaacqqG4baEcqqGTaqlcqqGXaqmaeaacqqGYaGmcqqGTaqlaaaaleqaaaqabeaacqqGtbWudaqhaaadbaGaemiEaGhabaGaeeOmaiJaeeyla0caaaaakiabbAeagjabbwgaLjabbofatnaaBaaaleaacqaIYaGmcqGGOaakcqWGZbWCcqGGPaqkaeqaaOGaaCzcaiaaxMaadaqadaqaaiabiodaZaGaayjkaiaawMcaaaaa@545C@

Furthermore, Berner described a magnetic, cubic iron monosulfide in pyrite forming environments [14,15], which was later identified as greigite (Fe_3_S_4_, the thiospinel of iron) [16]. Wilkin and Barnes [17] and later Benning et al. [18] presented evidence from laboratory experiments that greigite was implicated in the formation of pyrite from iron monosulfide precursors. However, it could not be resolved whether greigite was a true intermediate, and its kinetics and mechanism of formation or stability are still unknown. Greigite is being identified increasingly as an authigenic magnetic mineral in anoxic sediments due to its characteristic magnetic signature [19-21], and, together with hexagonal pyrrhotite, it is used as a paleomagnetic indicator [21]. The paleomagnetic use of greigite requires exact information on the kinetics and mechanism of its formation, and the conditions under which it is preserved over geological timescales.

Greigite is also synthesized by some bacteria [22-25]. Nanometer-sized greigite crystals form magnetosomes that cause these magnetotactic bacteria to be oriented in magnetic fields.

The investigation of the formation of pyrite from precursors such as mackinawite (FeS) and greigite is hampered by the fact that these phases are poorly crystalline and extremely sensitive to oxidation, which makes characterization by conventional powder X-ray diffraction (XRD) difficult. Furthermore, no wet-chemical technique is available for the distinction between and quantification of both phases, and they are commonly subsumed in the pool of "acid-volatile sulfide" (AVS) [13].

From a theoretical point of view, greigite can form on the pathway to pyrite as an intermediate species in the reaction between mackinawite and excess sulfur (Equ. 4) or via an iron loss pathway (Equ. 5).

3FeS→−2e−+S2−Fe3S4→+2S2−−4e−3FeS2     (4)
 MathType@MTEF@5@5@+=feaafiart1ev1aaatCvAUfKttLearuWrP9MDH5MBPbIqV92AaeXatLxBI9gBaebbnrfifHhDYfgasaacH8akY=wiFfYdH8Gipec8Eeeu0xXdbba9frFj0=OqFfea0dXdd9vqai=hGuQ8kuc9pgc9s8qqaq=dirpe0xb9q8qiLsFr0=vr0=vr0dc8meaabaqaciaacaGaaeqabaqabeGadaaakeaacqqGZaWmcqqGgbGrcqqGLbqzcqqGtbWudaWfqaqaamaaxacabaGaeyOKH4kaleqabaGaeyOeI0IaeGOmaiJaemyzau2aaWbaaWqabeaacqGHsislaaaaaaWcbaaccaGae83kaSIaee4uam1aaWbaaWqabeaacqaIYaGmcqGHsislaaaaleqaaOGaeeOrayKaeeyzau2aaSbaaSqaaiabbodaZaqabaGccqqGtbWudaWgaaWcbaGaeeinaqdabeaakmaaxacabaWaaCbeaeaacqGHsgIRaSqaaiabgUcaRiabikdaYiabbofatnaaCaaameqabaGaeGOmaiJaeyOeI0caaaWcbeaaaeqabaacciGae4NeI0IaeGinaqJaemyzau2aaWbaaWqabeaacqGFsislaaaaaOGaee4mamJaeeOrayKaeeyzauMaee4uam1aaSbaaSqaaiabbkdaYaqabaGccaWLjaGaaCzcamaabmaabaGaeGinaqdacaGLOaGaayzkaaaaaa@5729@

4FeS→−2e−−Fe2+Fe3S4→−Fe2+−2e−2FeS2     (5)
 MathType@MTEF@5@5@+=feaafiart1ev1aaatCvAUfKttLearuWrP9MDH5MBPbIqV92AaeXatLxBI9gBaebbnrfifHhDYfgasaacH8akY=wiFfYdH8Gipec8Eeeu0xXdbba9frFj0=OqFfea0dXdd9vqai=hGuQ8kuc9pgc9s8qqaq=dirpe0xb9q8qiLsFr0=vr0=vr0dc8meaabaqaciaacaGaaeqabaqabeGadaaakeaacqqG0aancqqGgbGrcqqGLbqzcqqGtbWudaWfqaqaamaaxacabaGaeyOKH4kaleqabaGaeyOeI0IaeGOmaiJaemyzau2aaWbaaWqabeaacqGHsislaaaaaaWcbaacciGae8NeI0IaeeOrayKaeeyzau2aaWbaaWqabeaacqaIYaGmcqGHRaWkaaaaleqaaOGaeeOrayKaeeyzau2aaSbaaSqaaiabbodaZaqabaGccqqGtbWudaWgaaWcbaGaeeinaqdabeaakmaaxacabaWaaCbeaeaacqGHsgIRaSqaaiabgkHiTiabbAeagjabbwgaLnaaCaaameqabaGaeGOmaidccaGae43kaScaaaWcbeaaaeqabaGae8NeI0IaeGOmaiJaemyzau2aaWbaaWqabeaacqWFsislaaaaaOGaeeOmaiJaeeOrayKaeeyzauMaee4uam1aaSbaaSqaaiabbkdaYaqabaGccaWLjaGaaCzcamaabmaabaGaeGynaudacaGLOaGaayzkaaaaaa@589D@

Sulfur-isotopic data presented by Wilkin and Barnes [17] indicate that even with an excess of sulfide in solution, the reaction proceeds via iron loss. However, these two pathways are stoichiometrically equivalent, because under non-sulfide-limiting conditions the Fe^2+^, which is released during the mackinawite transformation, is fed back into the reaction as newly formed nanocrystalline mackinawite. The reaction proceeding via sulfur addition (from dissolved zerovalent sulfur in the form of polysulfides, Equ. 6), allows the stoichiometry of electron transfer to be determined.

3FeS→Sx2--Sx-12-Fe3S4→Sx2-−Sx-22-3FeS2     (6)
 MathType@MTEF@5@5@+=feaafiart1ev1aaatCvAUfKttLearuWrP9MDH5MBPbIqV92AaeXatLxBI9gBaebbnrfifHhDYfgasaacH8akY=wiFfYdH8Gipec8Eeeu0xXdbba9frFj0=OqFfea0dXdd9vqai=hGuQ8kuc9pgc9s8qqaq=dirpe0xb9q8qiLsFr0=vr0=vr0dc8meaabaqaciaacaGaaeqabaqabeGadaaakeaacqaIZaWmcqqGgbGrcqqGLbqzcqqGtbWudaWfqaqaamaaoqcabaWccqqGtbWukmaaDaaameaacqqG4baEaSqaaWGaeeOmaiZccqqGTaqlaaaabeGccaGLsgcaaSqaaiabd2caTiabbofatPWaa0baaWqaaiabbIha4jabb2caTiabbgdaXaqaaiabbkdaYiabb2caTaaaaSqabaGccqqGgbGrcqqGLbqzdaWgaaWcbaGaeG4mamdabeaakiabbofatnaaBaaaleaacqaI0aanaeqaaOWaaCbeaeaadaGdKaqaaSGaee4uamLcdaqhaaadbaGaeeiEaGhabaGaeeOmaiJaeeyla0caaaWcbeGccaGLsgcaaSqaaiabgkHiTiabbofatPWaa0baaWqaaiabbIha4jabb2caTiabbkdaYaqaaiabbkdaYiabb2caTaaaaSqabaGccqqGZaWmcqqGgbGrcqqGLbqzcqqGtbWudaWgaaWcbaGaeeOmaidabeaakiaaxMaacaWLjaWaaeWaaeaacqaI2aGnaiaawIcacaGLPaaaaaa@5C7D@

In order that greigite is formed, three equivalents of FeS have to react with one equivalent of zerovalent sulfur (33 mol-% of zerovalent sulfur as polysulfide), while the complete reaction to pyrite requires another two equivalents of zerovalent sulfur (66 mol-% zerovalent sulfur) to reach completion. Overall, one equivalent of zerovalent sulfur per equivalent of FeS is required for the complete transformation to pyrite. Yet the conditions that control the reactions and kinetic parameters that govern greigite formation and stability have not been quantified.

In order to address these issues, hydrothermal experiments that followed the formation and transformation of crystalline phases on the pyrite pathway *in-situ *and in real time using synchrotron-based energy-dispersive X-ray diffraction (ED-XRD) have been carried out. The results presented below provide first quantitative insights into the kinetic parameters and activation energies of greigite and pyrite formation in anaerobic environments and in equilibrium with the reacting solutions. The results show that greigite is formed as an intermediate in the solid-state transformation of mackinawite to pyrite and that it plays a crucial role on the pyrite formation pathway. In addition, the parameters that control its stability and thus its preservation in the geologic record are discussed.

## Materials and methods

### Sample preparation

All reagents were prepared from analytical purity chemicals and deionized (DI) water (≥18 MΩ) that had been boiled for 30 minutes and cooled while being purged with oxygen-free nitrogen gas (C.P. grade, BOC gases). In addition, at all times open solutions or solids were handled or manipulated in an anaerobic chamber (Coy, MI, U.S.A.) which was maintained anaerobic by using a hydrogen/nitrogen gas mixture (5 %/95%, BOC gases) and a Pd/Pt catalyst.

The initial nanocrystalline iron monosulfide precipitate was prepared at 25°C in a fully sealed 500 mL glass reaction vessel following the methods described by Benning and coworkers [18]. Briefly, a fully deoxygenated 0.1 *M *solution of Mohr's salt [(NH_4_)_2_Fe(SO_4_)_2_*6 H_2_O, pH = 3.6] was saturated with H_2_S gas (C.P. grade, 99.5 % H_2_S, BOC gases) and the iron was quantitatively precipitated as iron monosulfide by raising the pH of the solution to 6.5 via the addition of a degassed 1.0 *N *NaOH solution. The sealed reaction vessel containing the inky black suspension was transferred to the anaerobic chamber and concentrated by settling and decanting. Care was taken not to loose any of the solid material during decanting and this way a final concentration of 0.17 M FeS was obtained. In order to determine the mineral composition of the precipitate, an aliquot of the suspension was filtered through a 0.2 μm cellulose nitrate membrane, dried inside the anaerobic chamber and the powder mounted onto a specially designed anaerobic X-Ray diffraction (XRD) sample holder with Kapton^® ^windows for conventional Cu-K_α _XRD analysis. The XRD patterns of the precipitate exhibited broad peaks at 5.05, 2.97, 2.31, 1.84, 1.81 and 1.73 Å (JCPDF file 24-0073) and a high background, thus confirming that the precipitate consisted of nanocrystalline mackinawite.

Polysulfide solutions synthesized using a modified standard procedure [26,27] were produced from a weighed amount of elemental sulfur that was dissolved inside the anaerobic chamber in a degassed 1.0 *N *NaOH solution that had been saturated with pure H_2_S gas. This resulted in a bright red solution of pH 8.3 containing 0.30 *M *zerovalent sulfur and 1.0 *M *H_2_S.

Special silica ampoules were designed for the anaerobic and hydrothermal *in-situ *transformation experiments. The ampoules could be heat-sealed, could withstand hydrothermal temperatures and pressures (up to 200°C and saturated water vapor pressure, SWVP) and could maintain anaerobic conditions. They were 50 mm long, with a 15 mm outer diameter, and a wall thickness of 1.5 mm, containing a total volume of 3.5 ml. They also contained a pre-sealed PTFE magnetic stirring bar.

Samples for the *in situ *experiments were prepared via a two-stage freezing procedure to preclude any interaction between the iron monosulfide suspension and the polysulfide solution. In a first step the silica ampoules were filled with 1.25 (± 0.13) mmol of the FeS suspension by means of a syringe equipped with a 15 cm needle. The suspensions were concentrated to a thick paste by centrifugation (1 min, 3000 rpm) and the excess solution was removed using a syringe. After evacuating using a hand pump, the ampoules were capped to preserve anaerobic conditions, removed from the anaerobic chamber and flash frozen in liquid nitrogen. In a second step, the frozen ampoules were reintroduced into the anaerobic chamber and 1.25 (± 0.13) mmol of zerovalent sulfur were added. The ampoules were again evacuated, flash frozen in liquid nitrogen, and immediately heat-sealed under vacuum using a glassblower's torch. The so prepared ampoules were kept under liquid nitrogen until the experiments were started.

### Energy-Dispersive X-ray Diffraction

The reactions between the nanocrystalline mackinawite suspension and the polysulfide solution were monitored *in situ *and in real time using the energy-dispersive X-ray diffraction (ED-XRD) setup of beamline 16.4 of the Synchrotron Radiation Source (SRS), Daresbury Laboratory [28-31]. Experiments were carried out at constant temperatures between 100 and 200°C and SWVP, and reactions were followed for up to five hours. At the start of a run, a freshly thawed ampoule was inserted into an aluminum heating block fitted with four resistance cartridge heaters and a thermocouple. The experimental charges were brought to the desired temperatures within 30 seconds (200°C) and 90 seconds (100°C, Benning unpublished results) after which the temperature controller and thermocouple maintained constant temperatures. Vigorous stirring ensured the sample's homogeneity and that the X-ray beam (1 mm diameter) passed through a representative sample of the ampoule's contents. White radiation produced by the 6 Tesla wiggler was passed through slits in the heating block and diffracted by the contents of the ampoules. A full diffraction pattern was collected as a function of energy simultaneously on three detectors set at 2θ angles of 2.92°, 5.44°, and 8.32°. The setting of the detector angles was chosen such that the growth and disappearance of non-overlapping peaks for each likely phase (mackinawite, greigite and pyrite) could be followed. The real time transformation reactions were observed by collecting a spectrum every 1 – 5 minutes. Due to interference of the beam with the heating block, in some experiments aluminum peaks were observed at 2.34 Å and 2.02 Å [(111) and (200), respectively].

### Data analysis

Using the software package X-fit [32], selected diffraction peaks of mackinawite, greigite and pyrite were fitted with peaks of Gaussian line shape. Peaks were chosen in a way that a) no peaks of different phases overlapped and b) a flat baseline could be fitted for at least 2 keV before and after the peak maximum. Distances of the crystal planes (d-spacings) were calculated from the fitted energy and the detector angle, and the crystalline phases were identified by comparison with their powder diffraction files (JCPDS International Center for Diffraction Data, 2001, mackinawite 24-0073, greigite 16-0713, pyrite 42-1340, Table 1).

**Table 1:** Assignment of Miller indices (hkl) to the peaks observed in Figure 1.

Peak Position (Å)	Assignment (hkl)^a)^		
	
	Mackinawite	Greigite	Pyrite
1.63			(311)* ^b)^
1.75		(440)*	
1.84	(200)*		
1.92		(511)	(220)
2.21			(211)
2.31	(111)		
2.42		(400)	(210)
2.71			(200)*
2.98	(101)	(311)	
3.13			(111)

a) JCPDS International Center for Diffraction Data, 2001: mackinawite 24-0073, greigite 16-0713, pyrite 42-1340.

b) marked peaks (*) were used to calculate the reaction progress variable α.

The position of the detectors prevented the evaluation of the strongest mackinawite reflection [(001), 5.05 Å]. The [(101), 2.97 Å] reflection for mackinawite was also not used due to its position on the crest of the background hump, and due to its overlap with the (311) peak for greigite (2.98 Å). In addition, the position of the greigite peaks (400) and (511) overlapped with the pyrite peaks (210) and (220), respectively and were thus not used. Lastly, the mackinawite (111) peak (2.31 Å) was also not utilized in quantitative evaluations due to the overlap with the (111) reflection of aluminum metal (2.34 Å). For these reasons, the (200) reflection of mackinawite, the (440) reflection of greigite and the (200) and (311) reflections of pyrite were used to quantify the progress of the reaction (Tab. 1).

The extent of the reaction (α) was determined by integrating the area under specific peaks and normalizing it to the maximum value (⟨ = 1) reached after the reaction was complete (pyrite) or to the initial value (mackinawite). Induction times were determined as the period before mackinawite started to disappear or greigite and pyrite started to appear. In the case of strong scattering of the initial or final values, an average α was determined from sections with no apparent change, i.e. during the induction time (mackinawite) and at the end of the reaction (pyrite), and normalized to α = 1. Fitting errors for each point were calculated as mean errors in α using an X-fit routine [32]. The time-dependent data were analyzed using a zero-order and a first-order kinetic model, and the Johnson-Mehl-Avrami-Kolmogorov (JMAK) [33,34] model.

Off-line experiments aimed at monitoring the changes in pH during the reactions, were carried out in a similar fashion at 40 – 100°C inside the anaerobic chamber in serum vials using an oil bath to control the temperature. The initial pH value of the reaction mixture (iron monosulfide suspension and polysulfide solution) was between 8 and 9 while in all experiments, the final pH value reached 9.0 on average (n = 14, max 10 days). At the end of the hydrothermal experiments, all ampoules were reintroduced into the chamber and broken open. The pH was measured and a final value of 9.6 obtained.

### Scanning Electron Microscopy

The suspensions were filtered (0.2 μm cellulose nitrate membranes) and the solid residue was washed three times with deoxygenated, DI water and dried at room temperature in the anaerobic chamber. The dried solid residues were re-dispersed in degassed 100% ethanol and a drop of the suspension deposited on an aluminum SEM stub. For SEM imaging the samples were coated with 3 nm of a Pd/Pt alloy and examined on a LEO 1500 series field emission gun scanning electron microscope (FEG-SEM) at 3 keV and a working distance of 3–6 mm.

### Transmission Electron Microscopy

In order to image the intermediate products and to document the existence of greigite as an intermediate phase, an experiment was prepared with 10 mol-% of zerovalent sulfur and reacted at 200°C. The reaction was monitored by ED-XRD as described above but this experiment was interrupted after 20 minutes, when greigite, but no other product had formed. The ampoule was flash-frozen in liquid nitrogen and stored frozen until it was broken open in the anaerobic chamber. The contents were filtered (0.2 μm cellulose nitrate membranes), washed three times with deoxygenated, DI water and dried at room temperature. The dried solid residue was dispersed in 100 % ethanol and one drop was deposited onto a standard holey carbon support film (Agar Scientific) inside the anaerobic chamber. The specimen was then mounted onto an anaerobic Gatan environmental transfer cell and transferred into the TEM without being exposed to the atmosphere.

The specimens were examined with a Philips CM200 Field Emission Gun -Transmission Electron Microscope (FEG-TEM) operating at 197 keV. The system was fitted with a Gatan Imaging Filter (GIF 200) and Oxford Instruments UTW ISIS X-ray detector (EDS) and EDS spectra were collected using a focused probe (5 nm diameter).

## Results and kinetic analysis

**Figure 1:**
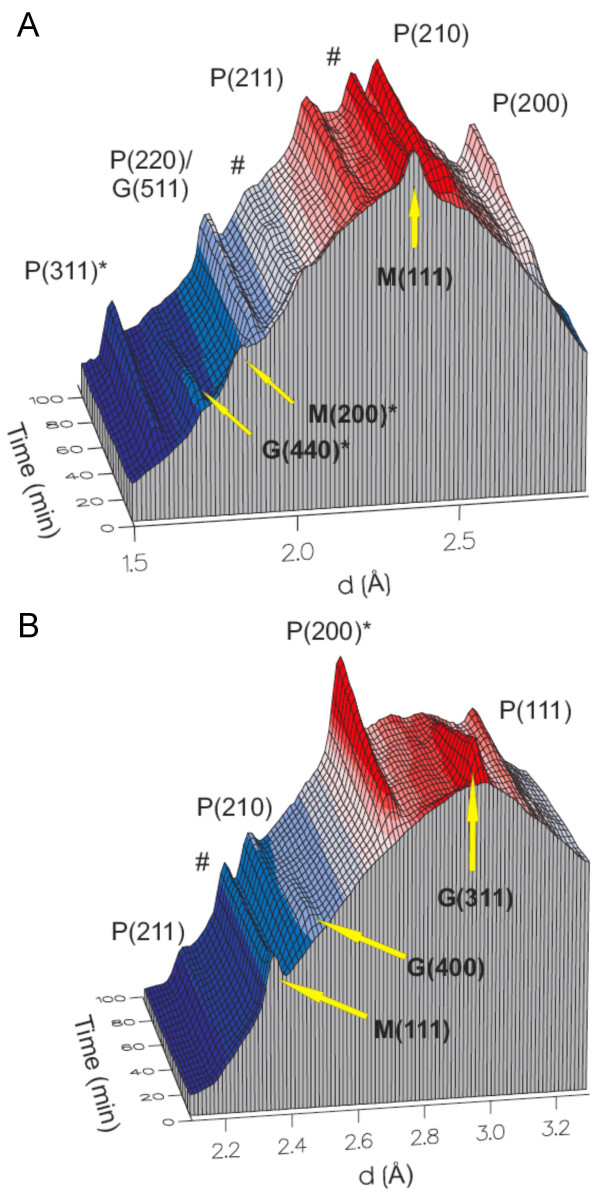
Three-dimensional representation of the time-resolved ED-XRD patterns from the A) top detector (8.32° 2θ) and B) middle detector (5.44° 2θ). Reaction conditions: 1.25 ± 0.13 mmol mackinawite, 1.25 ± 0.13 mmol zerovalent sulfur as polysulfide (100 mol-%, theoretically complete transformation to pyrite), 200°C. Miller indices and crystal lattice distances (d-spacings) are listed in Tab. 1. Peaks used for the determination of the reaction progress variable α are marked with an asterisk (*). Weak aluminum reflections [marked with #, 2.34 Å (111), 2.02 Å (200)] were observed because of interference of the beam with the aluminum heating-block.

The thawed, unreacted silica ampoules contained a voluminous black precipitate and a bright yellow supernatant, the color of which resulted from the polysulfide solution. After completion of the reactions, the supernatant was colorless and the volume of the solids was reduced from initially 50 % to approximately 20 % solid volume fraction, indicating an increase in crystallinity. Representative three-dimensional plots of the time-resolved diffraction data for the top and middle detector for an experiment at 200°C are shown in Fig. 1. Note that all initial diffraction patterns showed a broad background hump with only a few Bragg peaks for mackinawite, corresponding to the (111) and (200) crystallographic planes (2.31 Å and 1.84 Å, respectively). The broad background hump reflects the spectral intensity profile of the white beam scattered by the aqueous suspension in the ampoule [30].

**Figure 2:**
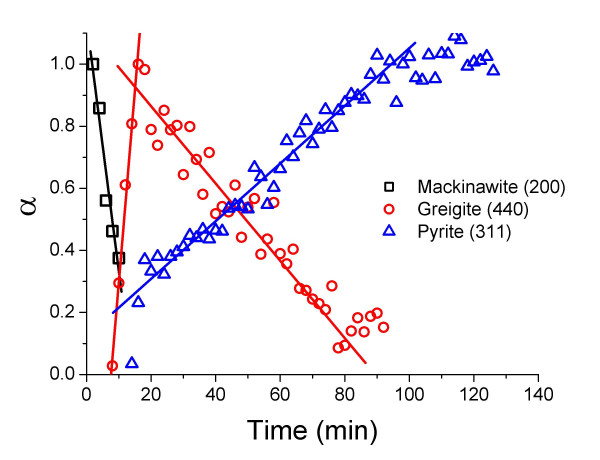
Reaction progress (α) determined from the growth and decay of the diffraction peaks for mackinawite (200), greigite (440), and pyrite (311) as a function of time at 200°C. Reaction conditions are the same as in Fig. 1. Rate constants for mackinawite consumption (k^-^_mack_), greigite growth and consumption (k^+^_grei _and k^-^_grei_, respectively) and pyrite growth (k^+^_pyr_) were determined from the slopes of the fitted lines and are tabulated in Tab. 3. Error bars were omitted for clarity; mean errors in α : mackinawite (200) ± 0.203, greigite (440) ± 0.0633, pyrite (311) ± 0.0489.

At temperatures between 125°C and 200°C mackinawite was transformed quantitatively to pyrite in the presence of 100 mol-% of dissolved zerovalent sulfur in the form of polysulfide. Greigite was observed as an intermediate phase at temperatures above 125°C. In Fig. 2 an example of the reaction progress at 200°C shows the disappearance of mackinawite, the appearance and subsequent disappearance of greigite as well as the growth of pyrite. Pyrite was the only product at completion of the reaction. Qualitatively it was observed that the diffraction peaks of greigite appear and grow as the diffraction peaks of mackinawite disappear, and the diffraction peaks of greigite and pyrite grow at the expense of those of mackinawite and greigite respectively.

In the specific case of greigite, evaluating the extent of reaction showed that with increasing temperature the area fits improved (resulting in less scattered values and reduced error in α) and this indicated that the crystallinity of greigite increased with temperature. Conversely, greigite was not observed below 150°C because the material was less crystalline and the amount of greigite in the beam path was not sufficient to produce a diffraction pattern.

In addition, as the temperature decreases, the induction times for the mackinawite disappearance and pyrite crystallization increase. Induction times were determined for mackinawite disappearance, greigite growth and decay, and pyrite growth by calculating the intersection of the linearly fitted line with α = 1 (mackinawite) or α = 0 (greigite and pyrite) (Table 2). Induction times for mackinawite consumption show the temperature behavior expected, i.e. they decrease with increasing temperature from immediate reaction at 200°C to 77 minutes at 100°C. Induction times for pyrite formation showed a similar behavior.

**Table 2:** Induction time and total reaction times, and final solid products of the hydrothermal transformation of mackinawite to pyrite ^a)^.

Temperature	Induction Times (min) ^b)^			Reaction Time (min)	Products (ED-XRD)
			
	t^-^_0 mack_	t^+^_0 grei_	t^+^_0 pyr_		
200°C ^c)^	0	8	0	90	Pyrite
200°C ^c)^	0	4	0	70	Pyrite, Greigite
175°C	7	n.a.^d)^	6	60	Pyrite, Greigite
150°C	4	6	12	74	Pyrite, Greigite
125°C	25 (± 3)	n.a.	22	110	Pyrite
100°C	77 (± 19)	n.a.	117 (± 30)	311 ^e)^	Mackinawite, Pyrite

a) All experiments: 1.25 ± 0.13 mmol FeS and 1.25 ± 0.13 mmol zerovalent S as polysulfide.

b) Mean errors in induction times, unless stated otherwise: ± 1. t^-^_0 mack_: induction time for mackinawite decay; t^+^_0 grei_, t^+^_0 pyr_: induction time for greigite and pyrite growth, respectively.

c) The reaction at 200°C was conducted in duplicate.

d) n.a.: not available.

e) Reaction was interrupted before completion.

Fig. 2 shows clearly that greigite was formed as an intermediate of the reaction, and that it was transformed to pyrite as soon as it was formed. The greigite maximum reached early in the reaction did therefore not correspond to a quantitative transformation of mackinawite to greigite, and the assignment of α = 1 to this maximum is therefore arbitrary. All kinetic parameters calculated for greigite formation or transformation were consequently overestimated. However, these figures clearly show that the growth of the greigite peak (440) corresponded with the decay/disappearance of the mackinawite peak (200) while in the second stage the decay/disappearance of the (440) greigite peak corresponds with the growth of the pyrite peaks (200) and (311).

**Figure 3:**
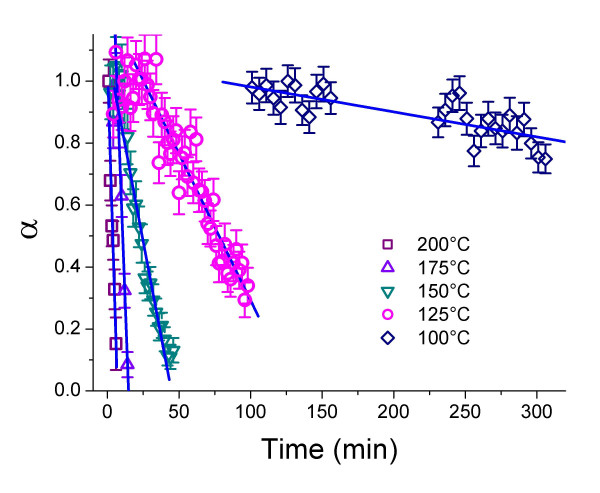
Reaction progress (α) following the decay of the mackinawite (200) diffraction peak area as a function of time and temperatures. Calculation of rates see caption Fig. 2.

Evaluating the changes in area under the mackinawite peak (200) shows that the decay of the mackinawite (200) peak follows a linear time dependence, indicative of zero-order kinetics of the reaction (Fig. 3). Conditional rate coefficients k^-^_mack_, which were defined following equation (7) were calculated from the slope of the fitted lines (Tab. 3), where the reaction progress variable α is the fraction of crystalline mackinawite remaining.

Rate=dαdt=kmack−α=1+kmack−⋅t     (7)
 MathType@MTEF@5@5@+=feaafiart1ev1aaatCvAUfKttLearuWrP9MDH5MBPbIqV92AaeXatLxBI9gBaebbnrfifHhDYfgasaacH8akY=wiFfYdH8Gipec8Eeeu0xXdbba9frFj0=OqFfea0dXdd9vqai=hGuQ8kuc9pgc9s8qqaq=dirpe0xb9q8qiLsFr0=vr0=vr0dc8meaabaqaciaacaGaaeqabaqabeGadaaakeaafaqaaeGabaaabaGaemOuaiLaemyyaeMaemiDaqNaemyzauMaeyypa0ZaaSaaaeaacqWGKbaziiGacqWFXoqyaeaacqWGKbazcqWG0baDaaGaeyypa0ZaaubmaeqaleaacqWGTbqBcqWGHbqycqWGJbWycqWGRbWAaeaacqGHsislaOqaaiabdUgaRbaaaeaacqWFXoqycqGH9aqpcqaIXaqmcqGHRaWkdaqfWaqabSqaaiabd2gaTjabdggaHjabdogaJjabdUgaRbqaaiabgkHiTaGcbaGaem4AaSgaaiabgwSixlabdsha0baacaWLjaGaaCzcamaabmaabaGaeG4naCdacaGLOaGaayzkaaaaaa@55D3@

The apparent activation energy of the transformation of mackinawite into greigite was calculated from the Arrhenius equation (Equ. 8).

kcond=A⋅exp⁡(−EaRT)     (8)
 MathType@MTEF@5@5@+=feaafiart1ev1aaatCvAUfKttLearuWrP9MDH5MBPbIqV92AaeXatLxBI9gBaebbnrfifHhDYfgasaacH8akY=wiFfYdH8Gipec8Eeeu0xXdbba9frFj0=OqFfea0dXdd9vqai=hGuQ8kuc9pgc9s8qqaq=dirpe0xb9q8qiLsFr0=vr0=vr0dc8meaabaqaciaacaGaaeqabaqabeGadaaakeaacqWGRbWAdaWgaaWcbaGaem4yamMaem4Ba8MaemOBa4Maemizaqgabeaakiabg2da9iabdgeabjabgwSixlGbcwgaLjabcIha4jabcchaWnaabmaabaWaaSaaaeaacqGHsislcqWGfbqrdaWgaaWcbaGaemyyaegabeaaaOqaaiabdkfasjabdsfaubaaaiaawIcacaGLPaaacaWLjaGaaCzcamaabmaabaGaeGioaGdacaGLOaGaayzkaaaaaa@477F@

[k_cond _(s^-1^): conditional rate constant, A (s^-1^): pre-exponential factor, E_a _(kJ mol^-1^): activation energy, R: universal gas constant, and T (K): absolute temperature.]

**Figure 4:**
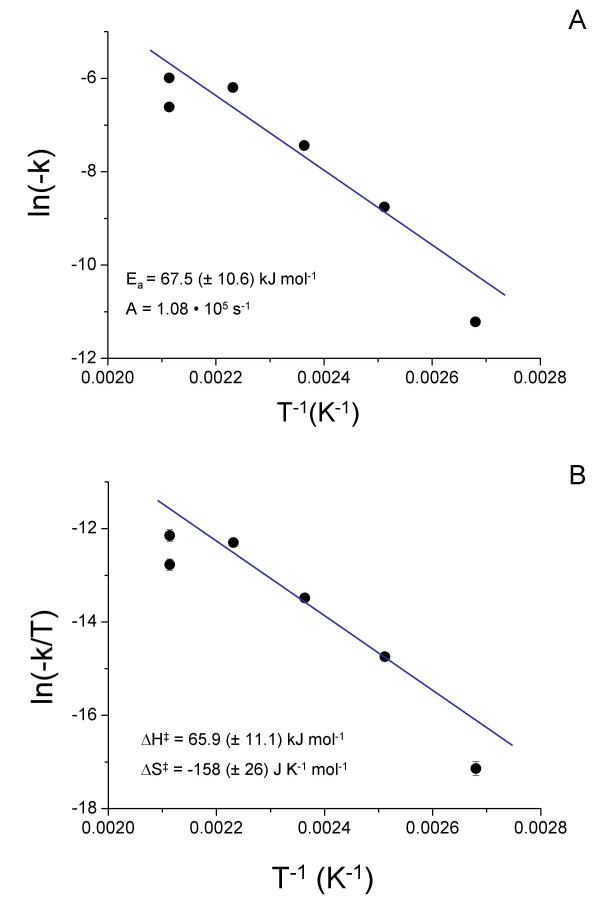
A) Arrhenius and B) Eyring plots of the rate constants calculated from the decay of the mackinawite (200) peak area at each temperature. Error bars are smaller than the dimension of the symbols.

Plotting ln (-k^-^_mack_) against T^-1 ^resulted in a straight line with slope -E_a_/R (Fig. 4A). The activation energy was calculated as E_a _= 67.5 (± 10.6) kJ mol^-1^.

The activation enthalpy was determined using the Eyring equation [34] (Equ. 9) and plotting ln (-k^-^_mack_/T) against T^-1 ^resulted in a straight line with the slope ΔH^#^/R (cf. Equ. 10 and Fig. 4B).

kcond=kTBhexp⁡(−ΔG‡RT)=kTBhexp⁡(−ΔH‡+TΔS‡RT)     (9)
 MathType@MTEF@5@5@+=feaafiart1ev1aaatCvAUfKttLearuWrP9MDH5MBPbIqV92AaeXatLxBI9gBaebbnrfifHhDYfgasaacH8akY=wiFfYdH8Gipec8Eeeu0xXdbba9frFj0=OqFfea0dXdd9vqai=hGuQ8kuc9pgc9s8qqaq=dirpe0xb9q8qiLsFr0=vr0=vr0dc8meaabaqaciaacaGaaeqabaqabeGadaaakeaacqWGRbWAdaWgaaWcbaGaee4yamMaee4Ba8MaeeOBa4Maeeizaqgabeaakiabg2da9maalaaabaGaem4AaS2aaSraaSqaaiabbkeacbqabaGccqWGubavaeaacqWGObaAaaGagiyzauMaeiiEaGNaeiiCaa3aaeWaaeaadaWcaaqaaiabgkHiTiabfs5aejabdEeahnaaCaaaleqabaGaeiyiGCiaaaGcbaGaemOuaiLaemivaqfaaaGaayjkaiaawMcaaiabg2da9maalaaabaGaem4AaS2aaSraaSqaaiabbkeacbqabaGccqWGubavaeaacqWGObaAaaGagiyzauMaeiiEaGNaeiiCaa3aaeWaaeaadaWcaaqaaiabgkHiTiabfs5aejabdIeainaaCaaaleqabaGaeiyiGCiaaOGaey4kaSIaemivaqLaeuiLdqKaem4uam1aaWbaaSqabeaacqGGHaYHaaaakeaacqWGsbGucqWGubavaaaacaGLOaGaayzkaaGaaCzcaiaaxMaadaqadaqaaiabiMda5aGaayjkaiaawMcaaaaa@645D@

ln⁡(kcondT)=ln⁡(kBh)−ΔG‡RT=ln⁡(kBh)+ΔS‡R−ΔH‡RT     (10)
 MathType@MTEF@5@5@+=feaafiart1ev1aaatCvAUfKttLearuWrP9MDH5MBPbIqV92AaeXatLxBI9gBaebbnrfifHhDYfgasaacH8akY=wiFfYdH8Gipec8Eeeu0xXdbba9frFj0=OqFfea0dXdd9vqai=hGuQ8kuc9pgc9s8qqaq=dirpe0xb9q8qiLsFr0=vr0=vr0dc8meaabaqaciaacaGaaeqabaqabeGadaaakeaacyGGSbaBcqGGUbGBdaqadaqaamaalaaabaGaem4AaS2aaSbaaSqaaiabbogaJjabb+gaVjabb6gaUjabbsgaKbqabaaakeaacqWGubavaaaacaGLOaGaayzkaaGaeyypa0JagiiBaWMaeiOBa42aaeWaaeaadaWcaaqaaiabdUgaRnaaBaaaleaacqqGcbGqaeqaaaGcbaGaemiAaGgaaaGaayjkaiaawMcaaiabgkHiTmaalaaabaGaeuiLdqKaem4raC0aaWbaaSqabeaacqGGHaYHaaaakeaacqWGsbGucqWGubavaaGaeyypa0JagiiBaWMaeiOBa42aaeWaaeaadaWcaaqaaiabdUgaRnaaBaaaleaacqqGcbGqaeqaaaGcbaGaemiAaGgaaaGaayjkaiaawMcaaiabgUcaRmaalaaabaGaeuiLdqKaem4uam1aaWbaaSqabeaacqGGHaYHaaaakeaacqWGsbGuaaGaeyOeI0YaaSaaaeaacqqHuoarcqWGibasdaahaaWcbeqaaiabcgcihcaaaOqaaiabdkfasjabdsfaubaacaWLjaGaaCzcamaabmaabaGaeGymaeJaeGimaadacaGLOaGaayzkaaaaaa@6597@

[k_cond _(s^-1^): conditional rate constant, k_B_: Boltzmann constant, h: Planck constant]

The activation enthalpy for greigite was calculated as ΔH^‡ ^= 65.9 (± 11.1) kJ mol^-1^, while the activation entropy was determined as ΔS^‡ ^= -158 (± 28) J K^-1 ^mol^-1^.

The two equations for the activation energy (Arrhenius and Eyring) are related via Equ. 11 [34] and testing this relationship for T = 473.15 K showed that the activation energy and the enthalpy are in agreement. The correction term *RT *is usually small compared to the activation enthalpy for the values and temperatures in this study. The activation energy is therefore approximately equal to the activation enthalpy. Similar to the correlation of activation enthalpy and energy, the activation entropy ΔS^‡ ^is related to the pre-exponential factor of the Arrhenius equation [34].

*E*_*a *_= Δ*H*^‡ ^+ *RT *    (11)

**Figure 5:**
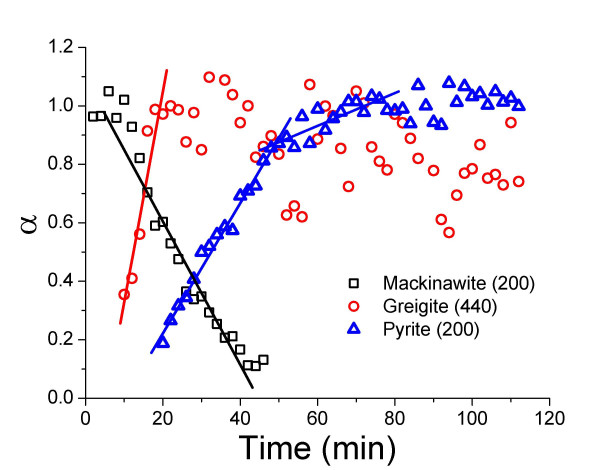
Reaction progress (α) determined from the growth and decay of the diffraction peaks for mackinawite (200), greigite (440), and pyrite (200) as a function of time at 150°C. Calculation of rates see caption Fig. 2. Error bars were omitted for clarity; mean errors in α : mackinawite (200) ± 0.0620, greigite (440) ± 0.115, pyrite (200) ± 0.0285.

Non-linear least-square regressions of the JMAK model [33,34] or a first-order model to the time-dependent data of greigite did not converge. Similar to the decay of mackinawite, the formation of greigite at 150 and 200°C followed zero-order kinetics (Fig. 5 and 2, respectively) and zero-order reaction rate coefficients were calculated by linear regression of the time-dependent data from the first occurrence of greigite to the maximum, which was set to α = 1 (Table 3). It is important to note that this maximum does not correspond to a complete transformation of mackinawite to greigite (as setting the maximum to α = 1 implies) and any rate constant derived for greigite was systematically overestimated. The greigite maximum coincided with the onset of pyrite formation (Fig. 2), indicating that the transformation to pyrite prevented the further growth of greigite. Below 200°C, mackinawite was still present when the maximum of greigite growth was reached, while at 200°C, the last occurrence of mackinawite and the onset of pyrite production coincided.

**Table 3:** Kinetic parameters determined from the reaction progress variable α.

Temperature (°C)	Rate constants (s^-1^)			
	
	k^-^_mack_	k^+^_grei_	k^-^_grei_	k^+^_pyr_
200^a)^	-1.35*10^-3^ (± 1.6*10^-4^)	2.07*10^-3^ (± 1.2*10^-4^)	-2.08*10^-5^ (± 9.7*10^-6^)	1.38*10^-4^ (± 4.9*10^-6^)
200^a)^	-2.50*10^-3^ (± 3.2*10^-4^)	4.75*10^-3^ (± 9.5*10^-4^)	-2.45*10^-4^ (± 6.7*10^-6^)	1.87*10^-4^ (± 5.0*10^-6^)
175	-2.05*10^-3^ (± 1.2*10^-4^)	n.a.^b)^	-3.33*10^-4^ (± 8.3*10^-5^)	3.72*10^-4^ (± 1.18*10^-5^)
				9.34*10^-5^ (± 1.57*10^-5^)
150	-5.90*10^-4^ (± 4.1*10^-5^)	1.20*10^-3^ (± 2.0*10^-5^)	-1.47*10^-4^ (± 3.3*10^-5^)	9.55*10^-4^ (± 1.62*10^-4^)
				5.5*10^-5^ (± 6.12*10^-6^)
125	-1.58*10^-4^ (± 8.3*10^-6^)	n.a.	n.a.	1.82*10^-4^ (± 1.12*10^-5^)
100	-1.35*10^-5^ (± 2.0*10^-6^)	n.a.	n.a.	8.15*10^-5^ (± 1.3*10^-5^)^c)^

a) The reaction at 200°C was conducted in duplicate. b) n.a.: not available. c) Reaction was interrupted before completion.

After reaching a maximum at an early stage in the reaction (e.g. after 20 min at 150°C, Fig. 5), greigite was slowly consumed reaching a constant value after approximately 90 minutes. Greigite reached relatively high final values for α at 150 and 175°C, but due to the arbitrary assignment of the maximum value to α = 1, the final greigite concentration is not necessarily as high as it appears. Zero-order rate coefficients of greigite decay were calculated by linear regression of the time-dependent data from the maximum value to the point where the constant final value was reached (Table 3).

**Figure 6:**
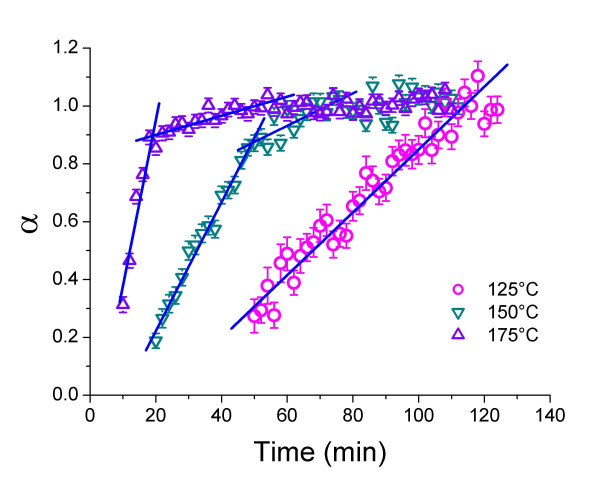
Reaction progress (α) determined from the growth of the pyrite (200) diffraction peak area as a function of time and temperature. Data for 100°C and 200°C are omitted for clarity. Calculation of rates see caption Fig. 2.

Similar to mackinawite consumption *vs*. greigite growth the reaction that describes greigite decay *vs*. pyrite growth respectively (after the greigite maximum) followed zero-order kinetics (Fig. 2 and 5). Contrary to the former, however, pyrite growth above 125°C and below 200°C was clearly biphasic and could be divided into two linear sections (Fig. 6; note that the pyrite data at 150 in Fig 5 is compared with data at 125 and 175 in Fig. 6). The boundary between these linear sections coincided with the full disappearance of mackinawite and thus with the maximum of greigite growth (Fig. 2 and 5). At 200°C, pyrite grew more slowly and its growth started only after mackinawite had been completely consumed and thus pyrite grew from the greigite intermediate in this stage.

Zero-order rate coefficients of this second phase of pyrite growth – corresponding to the consumption of greigite – were slower, whereas those of the first phase of pyrite growth were faster (e.g. at 175°C slow phase k = 9.35*10^-5 ^s^-1^, vs. fast phase k = 3.72*10^-4 ^s^-1 ^cf. Table 3).

Close inspection of Fig. 6 reveals that the transition between the first, fast phase and the second, slow phase of the reaction moves to earlier reaction times with increasing temperature. With this temperature behavior in mind, the slow pyrite growth at 200°C can be readily interpreted as the second growth phase. The transition between the fast and slow growth phase has therefore moved to the first pyrite appearance and explains the outlying first pyrite data point (Fig. 2).

**Figure 7:**
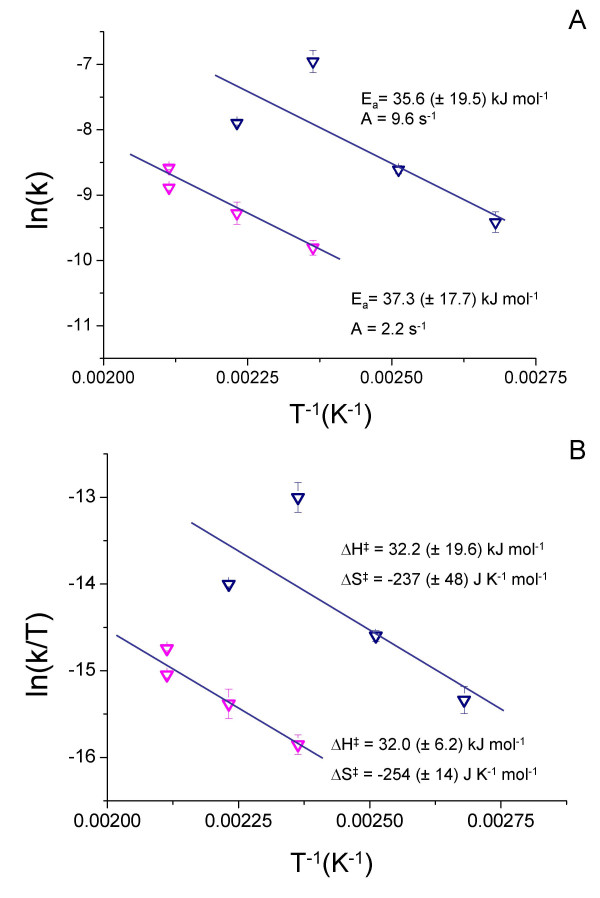
A) Arrhenius and B) Eyring plots of the rate constants calculated from the decay of the pyrite (200) peak area at each temperature.

Grouping the rate coefficients of the first (fast) phase of pyrite formation with those of pyrite formation below 150°C and the rate coefficients for the second phase (slow) with those of pyrite formation at 200°C, activation parameters were determined according to the Arrhenius (Equ. 8 and Fig. 7A) and Eyring equations (Equ. 9 and Fig. 7B). The linear regression lines for both phases of the reaction are parallel (within error) and the regression line for the faster phase was shifted to more positive values. The activation energy E_a _and the activation enthalpy ΔH^‡ ^for pyrite formation were therefore equal for both phases of the reaction and the only difference between the two reaction phases were the pre-exponential factor A (Equ. 8) and the activation entropy (Equ.9). The slower reaction phase exhibited a more negative activation entropy (-254 J K^-1^mol^-1 ^vs. -237 J K^-1 ^mol^-1^) and smaller value for A (2.2 s^-1 ^vs. 9.6 s^-1^), than the faster reaction phase. Note, however, that the uncertainty in the determination of the regression lines in Fig 7 created significant error in the pre-exponential factor and the activation entropy, respectively. Also note that the activation energy and enthalpy, and the pre-exponential factor for pyrite formation were smaller than the respective values for the mackinawite consumption, whereas the activation entropy was more positive (-158 J K^-1 ^mol^-1^).

## Discussion

The data shows that mackinawite disappeared as greigite grew, and that pyrite grew as greigite disappeared. This is strong evidence for greigite being an intermediate on the formation pathway of pyrite, and that pyrite did not form directly from mackinawite. In fact, the reaction profile shown in Fig. 2 is an example of the kinetics of a sequential reaction [35].

### The mechanism of the reaction mackinawite to greigite

The kinetics of the transformation of mackinawite to greigite and pyrite did not follow the model for the simultaneous nucleation and growth of crystals proposed first by Avrami [34,36-39] but rather obeyed a simple zero-order model. One corollary of this observation is that the transformations were kinetically neither limited by the concentration of polysulfide, nor by the amount of crystalline mackinawite or greigite. Both the polysulfide concentration and the mackinawite concentration decreased during the reaction. If these two parameters were kinetically limiting, this would result in first- or higher order dependence of the overall reaction on either quantity.

It has been shown by several researchers [7,12,28,31], that a surface-controlled dissolution/recrystallization mechanism follows a non-zero order rate law and is kinetically limited by the concentration of the solid educt. In the iron oxide system, the transformation of ferrihydrite to goethite has been shown to follow a dissolution/recrystallization mechanism [28,31,40,41], with a non-zero order rate law [28,31]. For iron sulfides, a dissolution/recrystallization pathway has been proposed for the direct formation of pyrite from FeS precursors via the H_2_S-pathway [10]. An equivalent mechanism has been suggested for the polysulfide-pathway [7] at 25 to 100°C. The rate limiting step of both the H_2_S-pathway and the polysulfide-pathway have been shown to be dependent on the concentrations of the precursor FeS and the oxidant (H_2_S and polysulfide, respectively) [7,12]. The zero-order kinetic model observed in this study for both the transformation of mackinawite to greigite and the transformation of greigite to pyrite under hydrothermal conditions above 100°C therefore requires an alternative mechanism than dissolution/recrystallization.

Lennie and coworkers [42] have studied the transformation of dry mackinawite to greigite using transmission electron microscopy. Based on their findings that the c-axes of associated mackinawite and greigite crystals were parallel (the a-axis of mackinawite formed a 45° angle with that of greigite) and that both structures are based on a cubic close-packed sub-lattice of sulfur atoms, they proposed a solid-state transformation mechanism. Their model requires the diffusion of approximately two of every four Fe^II ^cations from tetrahedral sites in mackinawite to octahedral sites in greigite with the concomitant oxidation of half of the migrating iron atoms to Fe^III ^resulting in an inverse spinel structure [13,43] (Equ. 12). They suggested that the excess Fe reacted with O_2 _or H_2_O at the greigite surface and that adsorbed O_2 _acted as electron acceptor [42].

4FeS→-2 e-,-FeIIFe3S4     (12)
 MathType@MTEF@5@5@+=feaafiart1ev1aaatCvAUfKttLearuWrP9MDH5MBPbIqV92AaeXatLxBI9gBaebbnrfifHhDYfgasaacH8akY=wiFfYdH8Gipec8Eeeu0xXdbba9frFj0=OqFfea0dXdd9vqai=hGuQ8kuc9pgc9s8qqaq=dirpe0xb9q8qiLsFr0=vr0=vr0dc8meaabaqaciaacaGaaeqabaqabeGadaaakeaacqqG0aancqqGgbGrcqqGLbqzcqqGtbWudaGdKaWcbaGaeeyla0IaeeOmaiJaeeiiaaIaeeyzau2aaWbaaWqabeaacqqGTaqlaaWccqGGSaalcqqGTaqlcqqGgbGrcqqGLbqzdaahaaadbeqaaiabbMeajjabbMeajbaaaSqabOGaayPKHaGaeeOrayKaeeyzau2aaSbaaSqaaiabbodaZaqabaGccqqGtbWudaWgaaWcbaGaeGinaqdabeaakiaaxMaacaWLjaWaaeWaaeaacqaIXaqmcqaIYaGmaiaawIcacaGLPaaaaaa@48E1@

Vaughan and Ridout [44] postulated that the extensive delocalization of Fe d-electrons in mackinawite leads to the formation of a conduction band, which would facilitate electron transfer to an adsorbed oxidant. In addition, mackinawite has been reported to readily reduce chlorinated hydrocarbons [45-48], and hexavalent chromium [4,5,49]. The reduction of the chlorinated hydrocarbons has been reported to proceed via electron-transfer at the mackinawite surface and was found to be complete within hours and days at room temperature [45,46,48].

Considering the electrophilic nature of the central sulfur atoms of the polysulfide anion [50], an electron-transfer from the mackinawite surface to adsorbed polysulfide followed by the atomic rearrangement in the solids is therefore proposed as the reaction mechanism under hydrothermal conditions. This is similar to the mechanism proposed by Lennie [42] for the dry transformation of mackinawite to greigite.

Another consequence of the zero-order kinetic model proposed here is that the rate-limiting step for the reaction can either be the electron-transfer from zerovalent sulfur to the mackinawite surface or the rearrangement of Fe atoms in the S sub-lattice, yet an unambiguous determination is not possible. The difficulty lies in the fact that both the adsorption of polysulfide to the mackinawite surface as well as the desorption of the polysulfide chain (reduced by one or more S-atoms) are fast equilibria compared with the electron transfer/atomic rearrangement.

### The activation parameters of greigite formation

Before discussing the activation energy, enthalpy and entropy of the greigite formation under hydrothermal conditions, it should be noted that the determined values are apparent activation parameters. These represent the contributions from various terms and not simply the potential barrier of an elementary reaction [34]. The activation parameters therefore merely provide one more line of evidence towards the reaction mechanism.

In all experiments the only intermediate observed in the mackinawite to pyrite reaction was greigite and the data strongly supports the direct transformation of mackinawite to greigite. The conditional activation enthalpy (65.9 kJ mol^-1^) and entropy (-158 J K^-1 ^mol^-1^) of mackinawite consumption are therefore the same as the activation enthalpy and entropy of greigite growth. Consequently, the rate constants of mackinawite consumption (k^-^_mack_) and greigite formation (k^+^_grei_) are equal. However, values of k^+^_grei _were consistently larger than the corresponding values of k^-^_mack_, e.g. at 150°C the value of k^+^_grei _was about twice that of k^-^_mack _(1.2*10^-3 ^and -5.9*10^-4^, respectively). This discrepancy can be explained by the fact that k^+^_grei _was systematically overestimated due to the arbitrary assignment of α = 1 to the maximum value.

The activation enthalpy of mackinawite formation under hydrothermal conditions (65.9 ± 11.1 kJ mol^-1^) is at the low end of the typically encountered range in solid-state reactions. Typically, the activation energies for bulk diffusion in the solid state lie between 20 and 80 kcal mol^-1 ^(approximately 80 – 320 kJ mol^-1^) [34]. The activation enthalpy, however, does not represent a solid-state diffusion process alone, but rather a combination of the initial electron-transfer reaction and the atomic movement. Oxidation of Fe(II) reduces the ionic radius of the Fe cation (from 78 pm in Fe^2+ ^to 65 pm in Fe^3+^), which destabilizes the crystal structure and facilitates the diffusion of the cation through the anion sub-lattice. The activation enthalpy of diffusion is therefore reduced compared with a purely diffusive process. The large negative value of the activation entropy (-158 ± 26 J K^-1 ^mol^-1^) is indicative of a large increase in order when the reaction reaches the transition state, or alternatively a very restricted reaction path [34], commensurate with the proposed diffusion of Fe cations through a limited number of possible paths in the sulfide sub-lattice.

**Figure 8:**
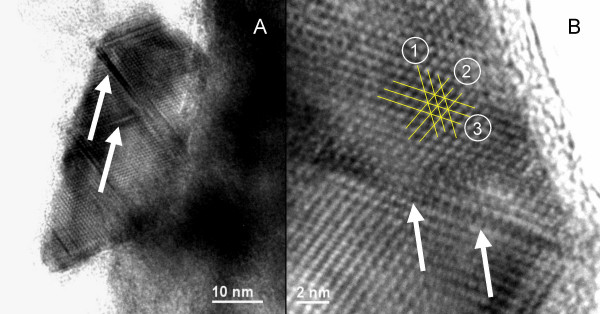
Typical HRTEM picture of greigite formed at 200°C in the presence of 10 mol-% of zerovalent sulfur as polysulfide and quenched with liquid nitrogen to -196°C. (A) The overview shows defects inside the crystal along crystallographic planes (arrows). (B) Three sets of lattice fringes are marked in the close-up: Set 1 has a distance of 0.56 nm, set 2 and 3 have a distance of 0.50 nm. Set 2 forms angles of 70° and 55° with sets 3 and 1, respectively. The distances and angular separations of the crystallographic planes are consistent with a greigite crystal viewed along the [01-1] crystal axis. Lattice fringe sets 2 and 3 correspond to the {111} plane and set 1 corresponds to the {200} plane. White arrows highlight a crystal defect causing a slight mismatch between adjacent lattices.

The morphology of greigite particles formed in these experiments supports the proposed solid-state mechanism (Fig. 8). Greigite particles formed at 200°C in the presence of 10 mol-% of zerovalent sulfur exhibit defects along crystallographic planes (Fig. 8a) which cause stacking faults and dislocations (Fig. 8b) [51,52]. These are similar to the structures observed in titanium dioxide nanoparticles that were grown by oriented aggregation at crystallographically specific surfaces, where small mis-orientations at the interface have led to twinning and dislocations [52,53].

The growth of minerals by oriented aggregation is a relatively new concept and little kinetic information is available [54-57]. Oriented aggregation describes growth by addition of solid particles to surfaces in a crystallographically controlled manner, which results in coherent interfaces and leads to the development of single homogeneous crystals [58]. This mechanism requires the fusion of the aligned crystal faces, e.g. by the condensation of nanocrystalline surfaces [59]. Penn developed a mechanistic model for oriented aggregation that is consistent with the kinetic data available [54]. It involved as a first step the reversible formation of a loose association of the nanoparticles, similar to an outer-sphere complex, which allows rotation to achieve the alignment of the corresponding surfaces. These surfaces connect in a second, irreversible step. This model treats nanoparticles as molecules and the reaction rate is therefore second order with respect to the concentration of the initial particles.

Huang and coworkers reported an activation energy (E_a_) of 136.8 ± 9.1 kJ mol^-1 ^for oriented aggregation of zinc sulfide nanoparticles coated with mercaptoethanol [56]. Although their kinetic data was in agreement with the proposed mechanistic model, Penn noted that the activation energy reported by Huang et al. very likely included an additional term for the desorption of mercaptoethanol and did therefore not represent an activation energy for oriented aggregation alone [54].

The defect features observed in hydrothermally grown greigite (Fig. 8) are in agreement with greigite growth by oriented aggregation of precursor mackinawite particles and consecutive oxidation and rearrangement of iron atoms. Mackinawite nanoparticles form a loose association by aligning crystallographically equivalent crystal faces. In a second step, mackinawite is oxidized by adsorbed polysulfide and iron cations migrate to the surface, where they react with excess sulfide in solution to form fresh mackinawite. This process also joins the aligned surfaces. Defects are created along the joints in the case of a slight mismatch of the aligned crystal faces, as seen in Fig. 8.

### The mechanism of the reaction greigite to pyrite

Greigite has been identified as an intermediate on the reaction pathway to pyrite by Wilkin and Barnes [17] and Benning and coworkers [18]. Wilkin and Barnes observed that the solid-state reaction of greigite to pyrite requires the outward diffusion of iron, the reduction of ferric iron and the oxidation of sulfide to disulfide [17]. They also presented strong evidence for a solid-state transformation (Equ. 13).

Fe3S4→−2e−,−FeII2FeS2     (13)
 MathType@MTEF@5@5@+=feaafiart1ev1aaatCvAUfKttLearuWrP9MDH5MBPbIqV92AaeXatLxBI9gBaebbnrfifHhDYfgasaacH8akY=wiFfYdH8Gipec8Eeeu0xXdbba9frFj0=OqFfea0dXdd9vqai=hGuQ8kuc9pgc9s8qqaq=dirpe0xb9q8qiLsFr0=vr0=vr0dc8meaabaqaciaacaGaaeqabaqabeGadaaakeaacqqGgbGrcqqGLbqzdaWgaaWcbaGaee4mamdabeaakiabbofatnaaBaaaleaacqqG0aanaeqaaOWaa4ajaSqaaiabgkHiTiabikdaYiabdwgaLnaaCaaameqabaGaeyOeI0caaSGaeiilaWIaeyOeI0IaeeOrayKaeeyzau2aaWbaaWqabeaacqqGjbqscqqGjbqsaaaaleqakiaawkziaiabbkdaYiabbAeagjabbwgaLjabbofatnaaBaaaleaacqqGYaGmaeqaaOGaaCzcaiaaxMaadaqadaqaaiabigdaXiabiodaZaGaayjkaiaawMcaaaaa@495F@

The zero-order kinetic model of pyrite formation from greigite suggested that, similar to the formation of greigite, the reaction was kinetically neither limited by the polysulfide concentration nor by the amount of crystalline greigite. In analogy to the formation of greigite it is therefore proposed that pyrite is formed via a similar sequence of adsorption of polysulfide followed by electron transfer and atomic rearrangement. In addition to the migration of one iron cation, the mechanism involves the oxidation of sulfide to disulfide and the reduction of Fe(III) to Fe(II) in the crystal [17]. The sulfide/disulfide redox couple has a more negative standard electrode potential in solution than the Fe (II/III) couple (-0.43 V and +0.771 V, respectively [60]), thermodynamically allowing the electron flow from sulfide to Fe(III). Standard electrode potentials in the solid state are not necessarily the same as in solution, but a complete reversal of the potentials appears unlikely. The electron transfer from sulfide to Fe(III) inside the crystal, and the formation of disulfide by distorting the sulfide sub-lattice to approach pairs of sulfur atoms, is therefore thermodynamically feasible. In summary, equ. 14 demonstrates the complex redox reactions (in the mineral phase) involved in the complete reaction pathway.

4Fe2+S2-→−2e−-Fe2+Fe23+Fe12+S42-→−Fe2+-2e-2Fe2+S21-     (14)
 MathType@MTEF@5@5@+=feaafiart1ev1aaatCvAUfKttLearuWrP9MDH5MBPbIqV92AaeXatLxBI9gBaebbnrfifHhDYfgasaacH8akY=wiFfYdH8Gipec8Eeeu0xXdbba9frFj0=OqFfea0dXdd9vqai=hGuQ8kuc9pgc9s8qqaq=dirpe0xb9q8qiLsFr0=vr0=vr0dc8meaabaqaciaacaGaaeqabaqabeGadaaakeaacqqG0aancqqGgbGrcqqGLbqzdaahaaWcbeqaaiabbkdaYiabgUcaRaaakiabbofatnaaCaaaleqabaGaeeOmaiJaeeyla0caaOWaaCbeaeaadaWfGaqaaiabgkziUcWcbeqaaiabgkHiTiabikdaYiabdwgaLnaaCaaameqabaGaeyOeI0caaaaaaSqaaiabb2caTiabbAeagjabbwgaLnaaCaaameqabaGaeeOmaiJaey4kaScaaaWcbeaakiabbAeagjabbwgaLnaaDaaaleaacqqGYaGmaeaacqqGZaWmcqGHRaWkaaGccqqGgbGrcqqGLbqzdaqhaaWcbaGaeeymaedabaGaeeOmaiJaey4kaScaaOGaee4uam1aa0baaSqaaiabbsda0aqaaiabbkdaYiabb2caTaaakmaaxacabaWaaCbeaeaacqGHsgIRaSqaaiabgkHiTiabbAeagjabbwgaLnaaCaaameqabaGaeGOmaiJaey4kaScaaaWcbeaaaeqabaGaeeyla0IaeeOmaiJaemyzau2aaWbaaWqabeaacqqGTaqlaaaaaOGaeeOmaiJaeeOrayKaeeyzau2aaWbaaSqabeaacqqGYaGmcqGHRaWkaaGccqqGtbWudaqhaaWcbaGaeeOmaidabaGaeeymaeJaeeyla0caaOGaaCzcaiaaxMaadaqadaqaaiabigdaXiabisda0aGaayjkaiaawMcaaaaa@6A22@

**Figure 9:**
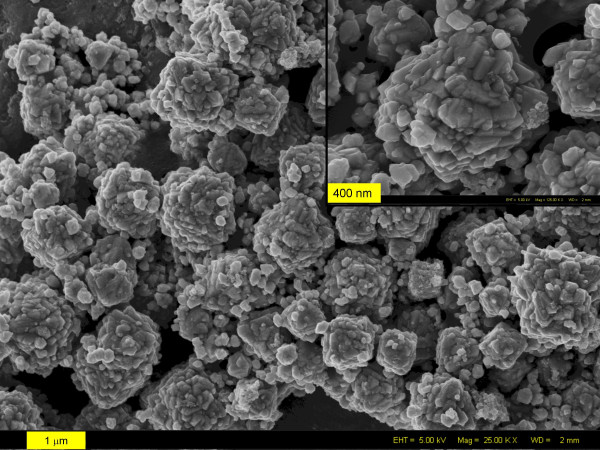
Typical FEG-SEM picture showing the octahedral morphology of pyrite grains formed at 200°C. Particles are 0.5 – 1.2 μm in size. Inset: Close-up of one particle showing the aggregation of smaller octahedra (50 – 100 nm).

The morphology of the pyrite crystals formed in the hydrothermal experiments supports the hypothesis of a solid-state transformation. Pyrite crystals of roughly octahedral shape (Fig. 9) were composed of smaller octahedra (inset of Fig 9), again indicating oriented aggregation as a likely mechanism for coarsening [52,53,58,59].

The reaction-order of the transformation of greigite to pyrite under hydrothermal conditions was not in agreement with that proposed for an oriented aggregation mechanism [54]. However, if the kinetically limiting step were not the association of the nanoparticles but rather the connection of the aligned particle surfaces, a reaction order independent from the particle concentration would be expected. The association and alignment of the greigite nanoparticles was facilitated by the magnetism of the particles, providing the fast equilibrium required by the fact that the association of particles was not kinetically limiting. Spontaneous aggregation of magnetic greigite particles has been observed before, e.g. in the magnetic ordering that has been suggested as a mechanism for the formation of pyrite framboids [61].

The connection of greigite particles to form pyrite required a different mechanism than the simple condensation observed for metal oxides. During the oxidation of greigite, Fe^2+ ^migrated outwards and reacted with the excess H_2_S in the system to form FeS, which in turn recrystallized to mackinawite and was oxidized to greigite and pyrite, thereby connecting the crystal surfaces.

The activation enthalpies of the transformation of greigite to pyrite (Fig. 7B slow and fast reaction) were indicative of a "surface-controlled" process rather than a solid-state diffusion process. Similar to the formation of greigite, however, the activation enthalpy represents the overall process instead of an elementary reaction. The combined reduction/oxidation processes destabilized the crystal, effectively lowering the activation barrier for the bulk diffusion process.

The temperature dependence of the pyrite formation kinetics, however, was dominated by the activation entropy, which had very large negative values (fast and slow phase of pyrite formation, respectively, Fig. 7B). These values reflected a large increase in order in the transition state. One contribution to this was clearly the arrangement of greigite nanoparticles in a way that led to coherent interfaces. Another important contribution to the activation entropy was the coordinated, spatially restricted movement of anions and cations in the crystal.

### Biphasic pyrite growth

The formation kinetics of pyrite were biphasic: Two linear rate constants could be fitted, one for the case when mackinawite was still present, and another when mackinawite had been consumed. While mackinawite was present, the formation kinetics of pyrite were faster (9.55*10^-4 ^s^-1 ^and 3.72*10^-4 ^s^-1 ^at 150 and 175°C, respectively) than when mackinawite had been used up (5.52*10^-5 ^s^-1 ^and 9.35*10^-5 ^s^-1^, respectively). The former values were within error equal to the rate of mackinawite consumption. While mackinawite was still present, the production of pyrite was kinetically limited by the consumption of mackinawite, respectively the production of greigite. Only after mackinawite had been used up was the reminder of greigite transformed as pyrite crystallized at a slower rate.

The biphasic growth of pyrite could be explained by considering the sequence of reactions leading to pyrite. Key property was the crystallinity of greigite: particle size and crystallinity of greigite increased with temperature, shown in the better developed diffraction peaks seen in the higher temperature experiments (cf. Fig. 2 and 5). The surface area, which decreased with increasing particle size, and the surface reactivity, which depends on crystal defects at the mineral surface, therefore decreased with increasing temperature. At higher temperatures, therefore, larger, better-developed crystals reacted via an oriented aggregation mechanism, requiring a larger degree of order.

This interpretation is supported by the activation parameters. The activation enthalpy of the aggregation of the smaller, less developed crystals is statistically not different from that of the larger, better developed crystals (Fig. 7B), indicating no change in the reaction mechanism. Due to the large error margins, the activation entropies of the two phases of the reaction are statistically not different. However, a trend to a more negative activation entropy for the slower reaction phase can be seen (Fig. 7B), which reflects the limited number of ways in which fewer, larger, and better crystallized particles can be arranged. In other words, oriented aggregation of smaller, less crystallized particles requires less ordering in the transition state and has therefore a more positive activation entropy. Note that because the mechanism of pyrite formation does not involve nucleation but rather proceeds via a solid-state transformation, induction times are not nucleation times.

## Implications

It is possible to estimate reaction times for pyrite and greigite formation in sedimentary environments using the activation energies determined in this study. For example, it can be calculated that the reaction time for the transformation of mackinawite to pyrite under non-sulfur limiting conditions is eight days at 10°C and twelve days at 4°C. It can be seen from the induction times listed in Tab. 2 that at these temperatures the induction times become more important. Using the data in Tab. 2, it can be estimated that the induction times add another 15 and 25 d to the reaction times at 10 and 4°C, respectively. At 25°C, the reaction time is reduced to 3 – 4 d, with the induction time adding 5 d.

These total reaction times (induction period and reaction time) are comparable to those reported in the literature for the reaction of iron monosulfide precursors with polysulfide [7,17]. Luther [7] reported reaction times of 7 to 15 d for the formation of pyrite from ferrous iron and polysulfide at 25°C. Wilkin and Barnes [17] found that a mixture of greigite and pyrite formed from mackinawite in the presence of polysulfide within 9 d at pH values between 7 and 8 and at 70°C. Hunger and coworkers [62] working under similar conditions (60 – 100°C and pH 8) followed the kinetics of the transformation of mackinawite to pyrite by quantifying the disappearance of polysulfide and found the reaction to be complete in 3 – 5 d.

## Conclusion

This research presents compelling evidence that greigite is formed as a true intermediate in the hydrothermal (100 – 200°C) transformation of mackinawite to pyrite using zerovalent sulfur as an oxidant. It has been shown that greigite forms readily in reactions of mackinawite and polysulfide at temperatures as low as 60°C [17,18,62]. With an excess of zerovalent sulfur, mackinawite is completely transformed to pyrite, whereas a mixture of mackinawite, greigite and pyrite are observed under sulfur-limited conditions [63]. The activation parameters determined in this study provide basic data for the estimation of reaction rates and reaction times in sedimentary environments. These results explain the widespread occurrence of authigenic greigite in sediments in association with both mackinawite and pyrite [1,2,13,21].
